# Comparing First-Year Engineering Student Conceptions of Ethical Decision-Making to Performance on Standardized Assessments of Ethical Reasoning

**DOI:** 10.1007/s11948-024-00488-y

**Published:** 2024-06-04

**Authors:** Richard T. Cimino, Scott C. Streiner, Daniel D. Burkey, Michael F. Young, Landon Bassett, Joshua B. Reed

**Affiliations:** 1 Otto H. York Department of Chemical and Materials Engineering, New Jersey Institute of Technology, Newark, NJ USA; 2 Department of Industrial Engineering, University of Pittsburgh, Pittsburgh, PA USA; 3 Department of Chemical and Biomolecular Engineering, University of Connecticut, Storrs, CT USA; 4 Learning Sciences Program, Neag School of Education, The University of Connecticut, Storrs, CT USA; 5 Department of Experiential Engineering Education, Rowan University, Glassboro, NJ USA

**Keywords:** Ethical reasoning, Concept maps, Engineering ethics, Ethical decision-making, Ethics assessment

## Abstract

The Defining Issues Test 2 (DIT-2) and Engineering Ethical Reasoning Instrument (EERI) are designed to measure ethical reasoning of general (DIT-2) and engineering-student (EERI) populations. These tools—and the DIT-2 especially—have gained wide usage for assessing the ethical reasoning of undergraduate students. This paper reports on a research study in which the ethical reasoning of first-year undergraduate engineering students at multiple universities was assessed with both of these tools. In addition to these two instruments, students were also asked to create personal concept maps of the phrase “ethical decision-making.” It was hypothesized that students whose instrument scores reflected more postconventional levels of moral development and more sophisticated ethical reasoning skills would likewise have richer, more detailed concept maps of ethical decision-making, reflecting their deeper levels of understanding of this topic and the complex of related concepts. In fact, there was no significant correlation between the instrument scores and concept map scoring, suggesting that the way first-year students *conceptualize* ethical decision making does not predict the way they behave when *performing* scenario-based ethical reasoning (perhaps more situated). This disparity indicates a need to more precisely quantify engineering ethical reasoning and decision making, if we wish to inform assessment outcomes using the results of such quantitative analyses.

## Introduction

Psychologists, ethicists and educators have been creating scenario-based instruments to assess moral development and ethical reasoning since at least the 1970s. Beginning with the Defining Issues Test (DIT) (Rest et al., 1974), Moral Judgment Interview (MJI) (Colby et al., 1987), and the updated form of the DIT—the DIT-2 (Rest et al., 1999), these instruments have been modeled to varying degrees on Kohlberg’s theory of moral development (Kohlberg & Hersh, 1977), which was conceived as a graduated scale of *stages and levels:* stages 1–2* (preconventional level),* stages 3–4 *(conventional level),* and stages 5–6 (*postconventional level*). The DIT-2 and similar instruments are designed to test the general population and have been applied to a variety of age groups and education levels. Within the past decade and a half, analogous instruments have been developed to assess distinct populations (Borenstein et al., 2008, 2010; Canny & Bielfeldt, 2016; Stransky et al., 2023). Of particular interest to our research is the engineering education-centric test called the Engineering Ethical Reasoning Instrument (EERI) (Zhu et al., 2014). Modeled on the DIT-2, the EERI was developed specifically to assess “individual ethical decision-making of engineering students in project-based design teams” (Zhu et al., 2014). Notably, the term *ethical decision-making* in the context of the EERI’s development is considered essentially synonymous with (or at least highly correlated to) *ethical reasoning*, though an individual student’s ability to connect these two concepts is an open question, one which is investigated in this paper. For this work, we define *ethical reasoning* as the active process of wrestling with an ethical dilemma: i.e., analyzing potential solutions, weighing their impacts on stakeholders, etc. When faced with an ethical dilemma and *actually making a choice*, we term it ethical decision-making. Naturally, ethical decision-making can be influenced by ethical reasoning, and as such the two concepts are linked. However, these concepts are not identical. The decision process reflects both the internal reasoning of the students and the nature of the environment (a situated, interactive, emergent view of ethical decision making). Furthermore, we posit that what these tests measure is *not* identical with most actual decision-making behavior as it occurs in lived experience, where individuals are plunged into complex, time-sensitive ethical situations in which personal risks and shared risks are in opposition, and decisions are often made based on incomplete knowledge and imperfect information (what they would do). In such situations, timely decisions are more likely to be made using intuition, self interests, and accumulated prior experience (Dreyfus & Dreyfus, 2004). Rather, these tests measure the abstract responses of individuals who are given time and opportunity to wrestle with issues and make well-thought-out decisions (what a reasonable, ethical person would do) at their leisure.

### Using the EERI as a Measure of Ethical Reasoning

The EERI has been used in numerous educational contexts to assess the ethical reasoning of engineering students, most frequently at the first-year undergraduate (Cimino & Streiner, 2018) and graduate levels (Hess et al., 2016; Kisselburgh et al., 2016). Research has focused primarily on the sensitivity of the instrument to engineering ethics education interventions in the short term (usually one semester) and is frequently paired with the DIT-2. The comparative research has focused on the relationship between population and P (Kohlbergian postconventional thought) and N2 (a mixture of the P score and the extent to which personal interest (I) and maintains norms (M) scores are eschewed) scores finding that in general, older and more experienced engineering students who take the EERI tend to score higher on the EERI than they do on the DIT-2, and when comparing scores pre- to post, the *changes* in N2 scores on the EERI tend to be larger and are more likely to be positive than the DIT-2 (Hess et al., 2016; Kisselburgh et al., 2016). This is attributed primarily to the more highly contextualized nature of the EERI, i.e., that when (mature) engineering students are faced with engineering ethics problems, they are more aware that it is best to take a principled perspective than an emotive or normative one. The reasons why this might be the case are complex and may have a lot to do with the differing sense of moral agency students feel in an engineering setting (such as those present in the EERI) versus more general settings (Cimino et al., 2022). Conversely, studies among first-year undergraduate students have presented evidence indicating ethics education might temporarily shift engineering students’ mindsets towards more conventional reasoning, i.e., lower P and N2 scores from pre to post (Cimino & Streiner, 2018). This situation may be the logical consequence of the juxtaposition of inexperienced engineering students—who are already more likely to be “conventional” thinkers than their graduate-level counterparts—with an ethics education environment where the students are learning to recognize and apply engineering codes of ethics for the first time. However, it is worth noting that in all cases above, the absolute value of the changes observed in DIT-2 or EERI scores assessed in pre-post fashion tend to be very small relative to the prevailing norms (Dong, 2009).

### Concept Maps as an Additional Assessment Tool

As discussed above, the DIT-2 and EERI are useful for evaluating a student’s ethical reasoning, both as a baseline measure and as a change-over-time measure. Yet, there is another critical piece to this learning puzzle: assessing prior knowledge related to ethics and ethical decision-making more broadly. Student-generated concept maps on ethical decision-making provide one such complementary measurement to ethical reasoning (i.e., comparing depth of ethical concept knowledge to ethical reasoning ability). Concept maps are tools “for people of all ages and all domains of knowledge to express their conceptual understanding about a topic” (Cañas et al., 2013). Concept maps have been found to be a useful tool for measuring conceptual knowledge on a topic of interest, as well as how that knowledge can change through instruction. Previous studies have utilized student-generated concept maps in areas such as engineering entrepreneurial mindset (Martine et al., 2019), sustainability knowledge (Watson et al., 2014), global workforce perceptions (Streiner et al., 2016), among others (Tan et al., 2017). Concept maps allow students to add any sub-concepts and make connections they deem relevant without prior guidance. This makes concept maps an ideal complementary assessment tool for investigating students’ conceptions of ethical decision-making.

### Statement of Intent

To date there has not been enough data collection using the EERI for any educational group to develop norms like those of the DIT-2, meaning that the values of the scores this instrument produces are not yet necessarily characteristic of particular educational groups (unlike the DIT-2, for which stable norms exist for all college education levels). For these reasons, the EERI can, and probably should continue to be paired with other instruments when assessing ethical reasoning until more data have been collected (Kisselburgh et al., 2016). Therefore the goal of this paper is to characterize the baseline ethical reasoning skills of first year (FY) undergraduate engineering students using both the DIT-2 & EERI instruments *and* baseline prior concept-knowledge in ethical decision-making using concept maps. It was hoped that this study will contribute to our overall picture of the ethical reasoning and ethical concept-knowledge of FY engineering students as a population. Furthermore, this study seeks to characterize the extent to which these disparate instruments correlate with one another, and to assess the relevance of the concept map as a tool for assessing ethics concept knowledge. This paper reports the findings from the DIT-2, EERI, concept maps, and their statistical relationship among first-year engineering students during their first academic year (Fall 2020–Spring 2021) of engineering education at the University of Connecticut (UConn), University of Pittsburgh (Pitt), and Rowan University (Rowan) as part of a larger project that investigated the effects of game-based instruction in engineering ethics education in the first year. We hope that in addition to helping build a body of literature that discusses alternative forms of assessment in engineering ethics, that it provides insights into the multifaceted nature of assessing ethical interventions, especially those rooted in active learning.

### Research Questions and Hypotheses

This paper explores the topics above by addressing the following research questions:

1. What are the baseline P/N2 scores of FY undergraduate engineering students who respond to the DIT-2/EERI, and how do they compare to the extant literature data for undergraduate students?
2. What is the nature of the relationship between ethical reasoning and ethical decision-making concept knowledge among FY engineering students?

It was hypothesized that baseline DIT-2 and EERI P/N2 scores of FY undergraduate engineering students would mirror those of the prevailing norms and existing small-scale studies, respectively. For students whose DIT-2/EERI scores reflected more postconventional levels of moral development and more “sophisticated” ethical reasoning skills, it was hypothesized that they would likewise have more detailed concept maps surrounding ethical decision-making (i.e., higher traditional and holistic scores), reflecting a deeper level of understanding of this topic.

## Methods

### Context of Study: Ethics Instruction Through Playful Learning

While it is not the goal of this paper to assess the effects of ethics instruction on instrument scores, it is still important to discuss the educational context in which this study was undertaken. Details of the ethics instruction framework and the educational contexts in which the instruments were deployed are described below.

### Educational Framework

Our framework for understanding all our playful learning activities in the classroom, as well as the ethical decision-making of engineers in authentic real-world settings, draws on the frameworks of embodied cognition (e.g., Barsalou, 2010; Muller, 2021), situated cognition (Brown et al., 1989; CTGV, 1990), and situated learning (e.g., Lave & Wenger, 1991). A situated cognition view of ethical decision-making also draws on the ecological psychology of Gibson (1986) and Dreyfus’ ethics of situated involvement, in which acting ethically is an acquired activity (2004). To understand behavior as “situated” is to reject a cognitivist description of thinking and knowing as storage and retrieval of concepts and schemas that reside in representational neural structures apart from the rest of our bodies, and adopts a dynamic description of knowing (and decision-making) as an emergent interaction between an intentional agent and an information-rich environment. Concepts then are indexical, taking on meaning only in the context in which they are used for intentional acts (see Shaw, 2001).

In our project this theoretical framework of embodied situated ethical decision making linked to several educational interventions designed to engage first-year engineers, in large lecture settings, in playful activities designed to make their learning more active. Our interventions used game mechanics and formats to capture student interest, engage them in peer interactions, and induce them to individually and collaboratively make decisions about engineering ethics topics by playing cards, predicting what other freshman classes might have decided, and to vote on how a choose-your-own Mars adventure would proceed week-to-week. These activities were specifically designed to alter student expectations about what they would be doing in a large lecture classroom, and to perhaps suspend their primary goals of achieving high grades, in favor of winning a game or enacting an interesting (funny, clever, creative) game strategy. This was intended to create a game situation, that while not authentic for the lived-in world of practicing engineers, might become an engaging situation in which students could explore their personal situated ethical decision making.

### Educational Contexts

At the University of Connecticut, three different game-based ethics education interventions were implemented in a first-year Foundations of Engineering course, which included students from all engineering disciplines except the computing majors. In the first intervention, students engaged weekly with an ethically-situated, narrative, choose-your-own adventure game that asked them to engage with an engineering contextualized story and then make ethically relevant decisions as well as respond to reflection questions. Students’ individual responses were aggregated and the decision with the most support was used to determine the direction of the story in the following week’s narrative. This activity took place over approximately twelve weeks of the Spring ‘21 semester. The other two interventions were an ethically situated card game and an ethically situated board game. Each was played in a single class period in week 9 and week 10 of the course.

Students at the University of Pittsburgh were exposed to the choose-your-own-adventure game across a 1 week period in a Spring ‘21 FY engineering course focused on computer programming. Students were asked to complete the game (and story) individually and at their own pace. Additionally, professional (and academic) integrity modules were included throughout the semester covering topics such as professionalism, academic dishonesty, and engineering ethical codes.

Finally, students at Rowan University completed all three game-based interventions (choose-your-own-adventure, ethically situated card game, and the ethically situated board game) across a subset of sections of a Spring ‘21 FY engineering course. The choose-your-own adventure game was implemented in a similar way as at the University of Connecticut.

### Data Collection: Defining Issues Test 2 (DIT-2) and the Engineering Ethical Reasoning Instrument (EERI)

To prevent survey fatigue and to ensure a roughly equal distribution of tests across the three institutions, the EERI was implemented at both the University of Connecticut and Rowan University, and the DIT-2 was implemented at both the University of Pittsburgh and Rowan University. The EERI resulted in a total of 425 students responding and the DIT-2 resulted in a total of 440 students responding. In all cases, students only took a single test regardless of their institution. The demographic distribution of these samples in terms of institution and self-reported sex is provided below (see Table 1). Ethical reasoning based on sex/gender/identity is not a main variable in this study, and as such this data is reported only for comparison with earlier studies, where modern notions of gender identity were not explicitly solicited (see Results and Discussion below).

**Table 1:** Self-reported sex* for EERI & DIT-2 administration

Institution	EERI (n = 425) (342 A, 83 C)		Institution	DIT-2 (n = 440) (331 B, 109 C)	
	Male (%)	Female (%)		Male (%)	Female (%)
UConn	64.0	36.0	Pitt	60.1	39.9
Rowan	78.3	21.7	Rowan	82.6	17.4

^*^Individuals were given the options of Female, Intersex, and Male for reporting purposes. There were no self-reported intersex individuals in this sample

### Data Collection: Concept Map Implementation and Assessment

University of Connecticut and Rowan University students were first given instruction in how to construct a concept map using the CMap software (Novak & Cañas, 2006) on a general topic to avoid bias and with the goal of ensuring familiarity with creating concept maps. Students were then asked to create their own concept map with the prompt “ethical decision-making” as their topic, with an allowance of 15–20 min. There were a total of 232 responses (198 at University of Connecticut and 34 at Rowan University), of which 225 were “usable” [i.e., created viable concept maps that could be measured using traditional scoring (Watson et al., 2016)]. The completed maps were first analyzed to quantify their level of complexity and sophistication by looking at the number of terms included, the number of hierarchies (levels of terms), and connections between them, termed “traditional scoring”. This traditional scoring analysis first appeared in the authors’ prior work (Reed et al., 2021), and is presented here for the purposes of comparison with the new analysis, termed “holistic scoring” (Besterfield-Sacre et al., 2004).

**Fig. 1:**
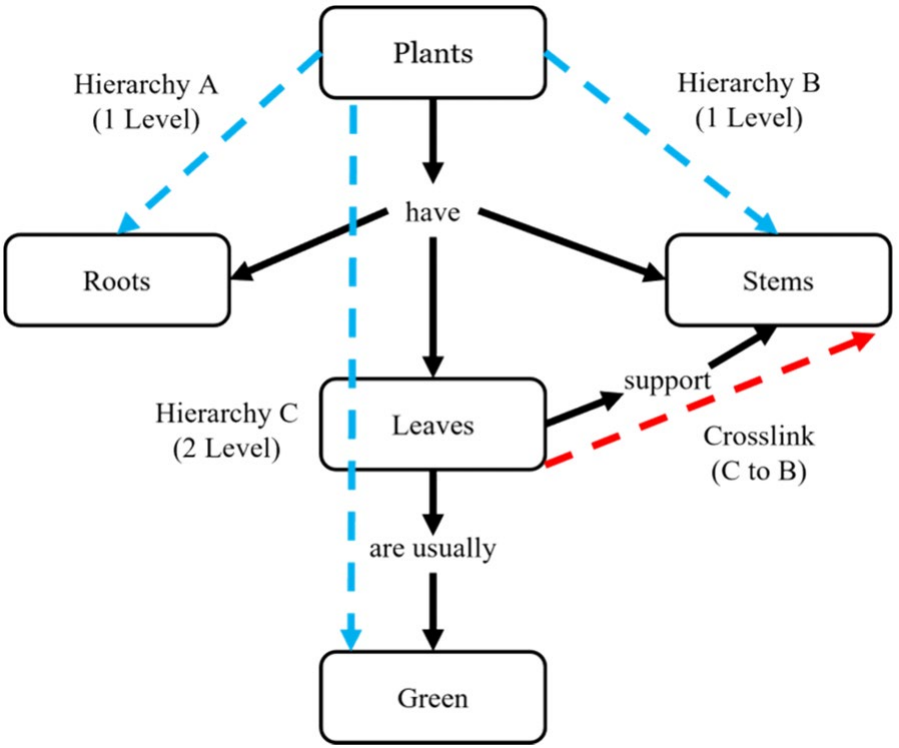
Breakdown of Concept Map (adapted from Watson et al., 2016)

Traditional scoring gives a concept map point values for the number of concepts (breadth of knowledge), number of hierarchies, the number of levels in the highest hierarchy (depth of knowledge), and the number of crosslinks (connectedness of knowledge). These components are detailed in Fig. 1 and through Eq. 1.

1 $${\text{Traditional Scoring}}.{\text{ Total}}\, = \,\left( {{\text{NC}}{-}{\text{NCL}}} \right)\, + \,\left( {{\text{HH}}} \right)\, \times \,{5}\, + \,\left( {{\text{NCL}}} \right)\, \times \,{1}0.$$

NC = Number of concepts, NCL = Number of crosslinks, HH = Highest level of hierarchy.

For Fig. 1 example (5 (NC)-3(NCL)) + 2(HH) × 5 + 3(NCL) × 10.

2 + 10 + 30 = 42.

In contrast, the holistic scoring method utilized a research-based Integrated Rubric for Scoring Concept Maps (IRSCM) to score based on comprehension, correctness, and organization (Besterfield-Sacre et al., 2004)—see Table 2. The IRSCM has been shown to be useful in capturing students’ conceptualizations of subject areas and can serve as an alternative to traditional scoring (Watson et al., 2016). As Besterfield-Sacre et al. (2004) write, *comprehensiveness* is used to determine the breadth and depth of students’ knowledge of a topic and how well they define the subject matter more broadly. *Organization* examines how students connect concepts and the logical approach for portraying these concepts. *Correctness* evaluates misconceptions students may have and the level of accuracy of the included concepts. Each rubric item was rated on a three-point scale (with 1 being low and 3 being high) and half-point scores were allowed.

**Table 2:** Holistic scoring rubric (Besterfield-Sacre et al., 2004)

	I	2	3
*Comprehensiveness *covering completely/broadly	The map lacks subject definition; the knowledge is very simple and/or limited. Limited breadth of concepts (i.e. minimal coverage of coursework, little or no mention of employment, and/or lifelong learning). The map barely covers some of the qualities of the subject area	The map has adequate subject definition but knowledge is limited in some areas (i.e., much of the coursework is mentioned but one or two of the main aspects arc missing). Map suggests a somewhat narrow understanding of the subject matter	The map completely defines the subject area. The content lacks no more than one extension area (i.e., most of the relevant extension areas including lifelong learning, employment, people, etc. are mentioned)
*Organization*to arrange by systematic planning and united effort	The map is arranged with concepts only linearly connected. There are few (or no) connections within/between the branches. Concepts are not well integrated	The map has adequate organization with some within/between branch coimections. Some, but not complete, integration of branches is apparent, A few feedback loops may exist	The map is well organized with concept integration and the use of feedback loops. Sophisticated branch structure and connectivity
*Correctness* conforming to or agreeing with fact, logic, or known truth	The map is naive and contains misconceptions about the subject area; inappropriate words or terms are used. The map documents an inaccurate understanding of certain subject matter	The map has few subject matter inaccuracies; most links are correct. There may be a few- spelling and grammatical errors	The map integrates concepts properly and reflects an accurate understanding of subject matter meaning little or no misconceptions, spelling/grammatical errors

**Fig. 2:**
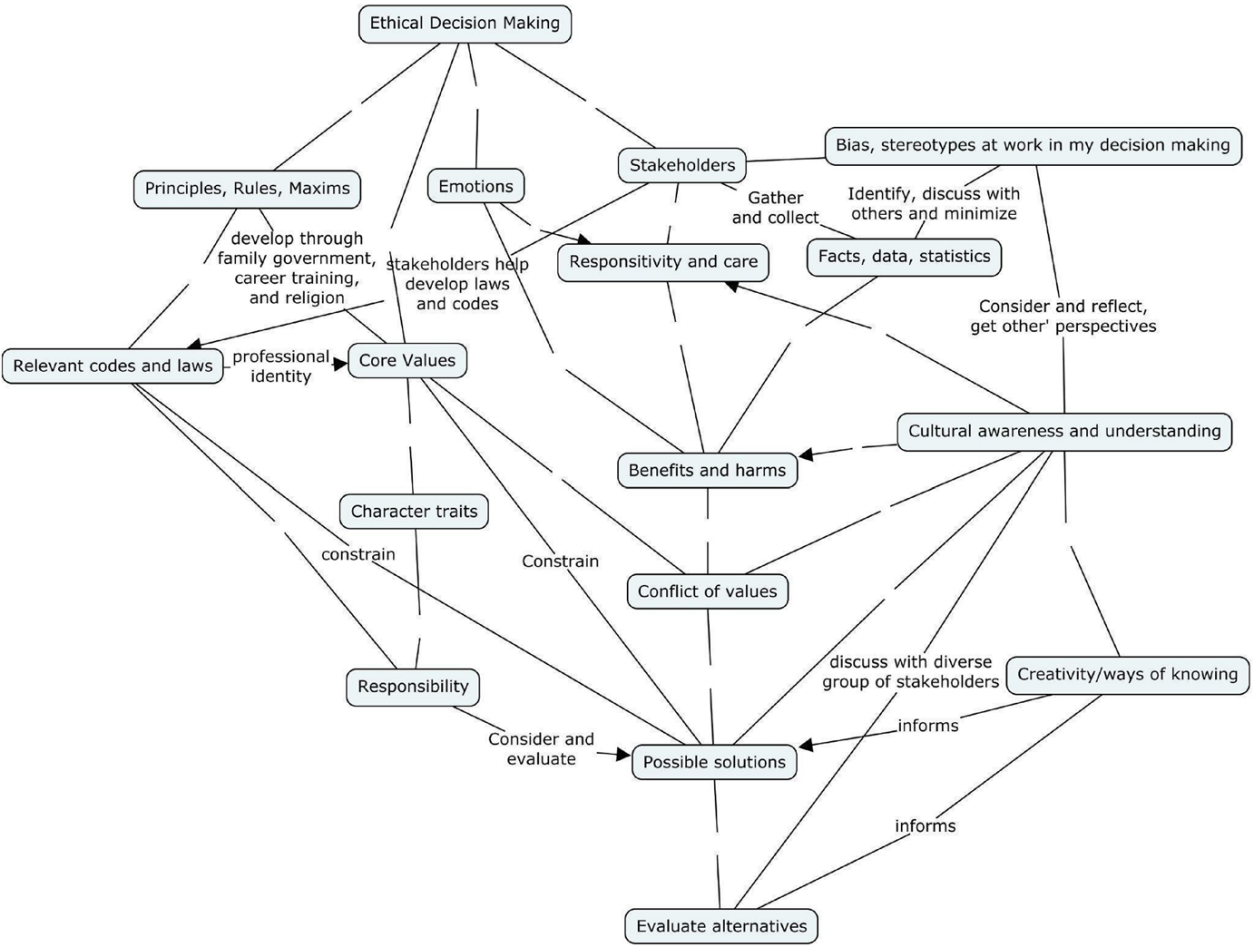
Expert Concept Map Developed via Modified Delphi Technique

The holistic scoring process started with two raters receiving instructions on how to assess concept maps using the IRSCM. First, the raters scored the same 5% of the concept maps (chosen randomly) and scores were compared. The intraclass correlation coefficient (ICC) was next calculated to determine interrater agreement. When the agreement was considered “good” (i.e. above 0.75) (Koo & Li, 2016), the maps were scored with remaining discrepancies being discussed amongst the research team in order to finalize the score. The raters were then given equal shares of the next 45% of the concept maps to be scored separately. The raters were then given the remaining 5% to score, share, and when reliability was strong enough, they finalized the scores in the same manner as above. More specifically, comprehensiveness was assessed based on the inclusion of sub-concepts that were included in an “expert concept map” (see Fig. 2). This was developed using a modified Delphi technique in which the research team (all who have taught, researched, and/or engaged in ethics education for several years) generated a list of concepts related to ethical decision-making and created a draft concept map together. This concept map was reviewed and revised by a Ph.D. trained ethics education consultant and sent back to the research team. This process was iterated several times until a final “expert concept map” was determined for use with the IRSCM. Organization was evaluated based on the types of connections students were making and whether they branched out linearly or utilized loops. Correctness was scored based on whether the concepts and connections between them were logical.

### Qualifications and Limitations

While the absolute quantities of student respondents in this study are substantial, it is also important to make note of several qualifying and limiting factors within the experimental design. Notably, all three of the universities involved in this study are major R1 universities in the Northeastern United States with diverse student populations, and the courses in which the students took the three assessments were all two-semester first-year undergraduate introductory engineering design course sequences: one lab based (Rowan University) and others lecture based (University of Connecticut, Pittsburgh). In most instances, the data collection was performed by a PI who also taught the course, though in some cases additional volunteer instructors were involved as well—particularly at Rowan University. While the students in all cases were provided with a brief video training on concept map creation, there was natural variability in the relative emphasis instructors placed on the concept map assignment. Likewise, this study also took place during the height of the COVID-19 pandemic, with Fall’20 courses being largely synchronous online, and Spring’21 courses a vacillating mixture of face-to-face and synchronous online instruction. As such, student engagement and persistence were somewhat lower than expected in a typical year. Finally, it is important to state that the EERI is still in the process of validation, though notably this study took place after several recent changes were implemented to improve the test’s validity after partial confirmatory factor analysis (Odom, 2020; Odom & Zoltowski, 2019). As such, the absolute numerical values of the present EERI test scores may not be directly comparable to prior published work. Likewise, EERI response data has not yet been benchmarked to the extent that the DIT-2 has, and so it is not currently possible to tell whether the test is sensitive to education level in a similar manner as the DIT-2.

## Results and Discussion

### Assessment of Ethical Reasoning Using EERI and DIT-2

The mean of the DIT-2 P and N2 scores were compared to the DIT-2 norms of first-year college students generated by the University of Alabama’s Center for the Study of Ethical Development, presented in Table 3. The DIT-2 norms represent collected responses from 652 diverse data sets from 2005 to 2009 that contain students from many different majors, disciplines, and areas of study (Dong, 2009). The student scores from the present study were statistically greater than the DIT-2 norms for first-year students (Dong, 2009). The EERI statistics are also compared to the pretest scores of a previous study of first year engineering students at Rowan University in Table 3 (Cimino & Streiner, 2018) in which FY engineering students in the same course as the present study took the DIT-2 or the EERI in a pre-post fashion, with a traditional set of ethics interventions in between. In that small-scale study, it was found that there was no statistically significant change in P/N2 scores from pre-to-post, signaling that these instruments may not have the resolution to detect changes in ethical reasoning across a single semester for the FY population (or in the worst case, that the ethics interventions were not capable of shifting student behavior towards postconventional reasoning). Like with the DIT-2, both the P and N2 student scores of the EERI are slightly higher but generally consistent with the previous study.

**Table 3:** DIT-2 and EERI baseline scores compared to previous data (Dong, 2009; Cimino & Streiner, 2018)

DIT-2 scores	Present study (n = 440)	DIT-2 norms (n = 10,319)	2-sample T-test	
	Average	Average	*p* value	Cohen’s D (effect size)
P	36.62	34.11	< 0.001	0.168
N2	35.29	33.42	0.012	0.128

EERI scores	Present study (n = 425)	EERI prior study (n = 34)	2-sample T-test	
	Average	Average	*p* value	Cohen’s D (effect size)
P	57.50	56.56	0.770	0.053
N2	54.71	54.32	0.905	0.022

The EERI and DIT-2 scores were then disaggregated into groups based on self-reported sex. Females scored significantly higher on both the P and N2 scores for the DIT-2 than did males, which is in line with previous research (Bebeau, 2002; Bielby et al., 2011; Maeda et al., 2009). Females scored significantly higher on both the P and N2 scores for the EERI as well, but with a lower effect size. The difference in ethical reasoning between male and female students that is found to be significant for both P and N2 scores of the DIT-2 is consistent with previous research. In particular, it has been previously found that females both within and outside of the field of engineering score significantly higher on the DIT-2 than do males (Bebeau, 2002; Bielby et al., 2011; Maeda et al., 2009). Likewise, in previous research using the EERI, females scored consistently higher than males, but this difference was not statistically significant (Zhu et al., 2015). However, the current study found that females scored significantly higher on both the P and N2 measures for the EERI. It is worth noting here also that the scores of the EERI in the current study align closely with the *post*-scores of higher-educated engineering students assessed in another unrelated study (Kisselburgh, 2016). However, as mentioned above in the Limitations, it is impossible to draw any firm conclusions about this observation since the EERI itself is not yet sufficiently benchmarked to allow direct comparison among students at different education levels.

### Concept Map Scoring

The concept maps that the first-year engineering students created in their respective courses were scored both through the process of traditional scoring and holistic scoring. Traditional scoring was accomplished using the CMapParse program and the scores were then compiled in SPSS Statistics (Watson et al., 2018). Traditional scoring found the number of concepts, number of hierarchies, highest level of hierarchy, number of crosslinks, and the total traditional score using the previously described variables (Table 4). The results of the traditional scoring were published in the authors’ previous work (Reed et al., 2021) and are reproduced below.

**Table 4:** Descriptive statistics for concept maps using traditional scoring (n = 225) from (Reed et al., 2021)

Variable	Average	SD
Number of concepts (NC)	16.30	7.25
Number of hierarchies	5.34	3.67
Highest hierarchy (HH)	3.38	1.74
Number of crosslinks (NCL)	1.77	3.50
Traditional score	49.15	39.40

*SD* standard deviation

The descriptive statistics in Table 4 display a large variation between students’ traditional scores and the associated variables (Traditional Score = 49.15 ± 39.40). Comparing the Number of Concepts to the other score variables (especially the Number of Crosslinks) it was found that students focus more on the concepts known (16.3), but rarely show how those concepts are interrelated to each other with crosslinks (1.77). Crosslinks are the weakest area of the concept maps with 68.9% having less than 2 crosslinks. Learning research has demonstrated that knowledge is not just about concept retrieval, but specifically drawing the connections between concepts (Rittle-Johnson, 2006; Star, 2005), and as such, these results are suggestive of a population which has limited reflexive knowledge of ethical decision-making. Such a population could be ripe for influence using tools to develop reflexive principlism such as those espoused by (Beever & Brightman, 2016).

**Fig. 3:**
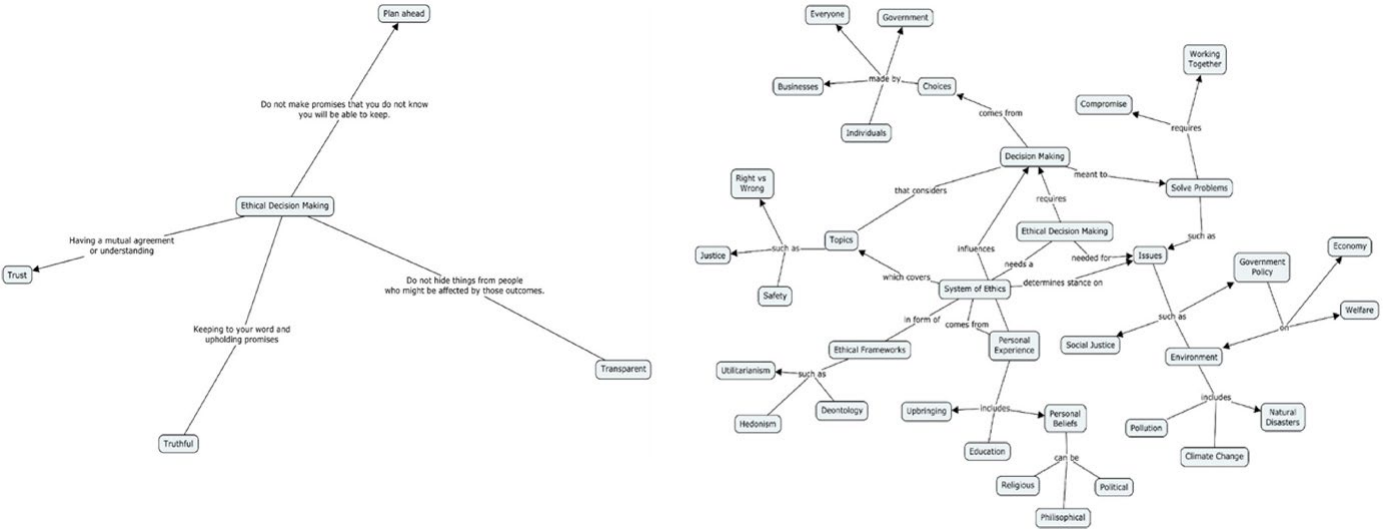
Example of bottom quartile map (left—Traditional Score = 9 pts) and top quartile map (right—Traditional Score = 110 pts). Figure reproduced from (Reed et al., 2021)

The concept maps were next divided up into quarters based on their traditional scores to explore the nature of the differences between the highest and lowest scoring maps (i.e., where is the biggest gap in terms of traditional scoring variables). Figure 3, reproduced from (Reed et al., 2021) illustrates two representative concept maps—one with a low traditional score from the lowest quartile (the bottom 25% of scores) and another with a high traditional score from the highest quartile (the top 25% of scores). Comparing the maps in Fig. 3, it is easy to see many of the differences between these groups: top-quartile maps have more concepts and a denser looking map with a higher number of cross-links, as well as more hierarchies that go to deeper levels.

Table 5 shows a more precise picture of how the average bottom quartile map compares to the average top quartile map. The coefficient of variation (CV) is the ratio of the standard deviation to the mean and was used to more easily compare the dispersion of data between the different variables. The top quartile maps had twice as many concepts, on average, as bottom quartile maps and likewise maintained a similar CV. Despite there being almost no difference in the number of hierarchies between the two quartiles, the top quartile maps illustrated that these individuals can draw deeper connections between associated concepts in ethical decision-making than bottom quartile individuals, with over twice as many levels of hierarchy as those of the bottom quartile. In fact, the bottom quartile maps do not include a single cross link. Furthermore, the average total scores between top and bottom quartiles are vastly different, with the highest scoring maps being almost five times higher than the low scoring maps. However, the CV in top quartile maps is almost double that of the bottom quartile, due to a small number of very high scores. Overall, the bottom quartile maps were more consistent in their map construction with lower variation, but top quartile maps had higher scores across the board, implying that top quartile individuals are in general substantially more capable of identifying ethical decision-making concepts and drawing connections between associated concepts than bottom quartile individuals.

**Table 5:** Descriptive Statistics for Low vs. High Traditional Scoring Concept Maps

Variable	Low traditional scoring (bottom quartile, n = 64)			High traditional scoring (top quartile, n = 64)		
	Average	SD	CV	Average	SD	CV
Number of concepts (NC)	10.14	3.29	0.32	21.80	7.93	0.36
Number of hierarchies	5.08	2.70	0.53	5.43	4.10	0.76
Highest hierarchy (HH)	1.98	0.49	0.25	5.20	1.92	0.37
Number of crosslinks (NCL)	0	0	N/A	5.33	4.94	0.93
Traditional score	20.06	4.40	0.22	95.77	46.40	0.48

*SD *standard deviation, *CV* coefficient of variation

The holistic scoring statistics also provided insight into the distribution of comprehension about ethical decision-making and concept organization among first-year students, resulting in an average total score of 5.40 out of 9.00 (see Table 6). The average comprehension and organization scores of the concept maps (1.60 and 1.77, respectively) were the weakest areas, with average correctness scores being somewhat higher. The low organization scores again exemplified the lack of crosslinks that show the interrelation of concepts. The low average comprehension score showed that in general first-year engineering students have a novice’s understanding of ethics prior to formal ethics education. While the average correctness score was higher than the others (2.03 out of 3), there was clearly still room for improvement. Taken together with the traditional scoring, these holistic scores start to paint a picture for how first-year engineering students initially approach ethical reasoning and ethical decision-making. While they may understand many concepts associated with ethics *in general*, they may not fully comprehend the relationships between these concepts or how they might weigh into their own ethical decisions in context (Detterman & Sternberg, 1993). This showcases the need for instructors to focus on the interrelation of topics in the engineering ethics classroom which can lead to a deeper understanding of ethics and ethical reasoning (Gauthier, 2013). Case studies and similar strategies, such as role-playing games based in case studies, have been shown to reinforce connections in many topics in engineering ethics such as analyzing situations, considering outcomes, acknowledging biases and values, implementing codes of ethics, and promoting an ethic of care (Hess & Fore, 2018; Loendorf, 2009). As such, the authors’ educational approach, incorporating context-rich role playing scenarios (Streiner et al., 2021), have the potential for such reinforcement.

**Table 6:** Holistic Scoring Summary (Minimum = 1, Maximum = 3)

Variable	Definition	Average	SD
Comprehension	Broadness and depth of knowledge	1.60	0.46
Correctness	Accuracy of information	2.03	0.50
Organization	Well-defined and logical branching	1.77	0.49
Holistic score	Summed scores	5.40	1.17*

^*^Holistic SD = standard deviation of holistic scores (i.e., not sum of SDs)

*SD *standard deviation

### Relationship Between Conceptualizations of Ethical Decision-Making (Concept Maps) and Performing Ethical Reasoning (DIT-2/EERI)

The results of the EERI and DIT-2 assessments suggest that engineering students come into their first-year engineering program with a level of ethical reasoning comparable to the population norm at this level of education. Furthermore, the concept map data indicates that they do not have as rigorous an understanding of ethical decision-making as more experienced engineering professionals (the authors). At a deeper level, it was also observed that first-year students do not fully understand the relationships between many of the concepts that they *do* know in ethics and ethical decision-making. This observation may be the product of students who are drawing more on their experiences of normative social ethics or personal ethics (Abaté, 2011) rather than professional ethics (Harris et al., 1996). These normative social and personal conceptions of ethics are often instilled within people from a young age by the people and culture surrounding them (Abaté, 2011), and as such become second nature, resulting in a diverse range of emotive, instinctive or intuitive responses to common ethical scenarios (Sadler & Zeidler, 2005). Professional ethics, however, are the agreed-upon standards that guide those who work in a specific field (Harris et al., 1996). It is logical that students at this level would react to a situation in engineering by drawing upon more normative social and personal experiences/instincts, but not professional ones, due to their relative inexperience. Notably, engineering ethics codes are a major part of first-year engineering ethics education, and so recognition of the professional dimensions of ethics would be expected to change post-intervention. Yet, none of this discussion precludes the fact that first-year engineering students do often have their own preconceived notions of what an engineer is “supposed” to do in a given situation (Cimino et al., 2022) regardless of whether these opinions concur with actual professional codes (Davis, 1999). However, when students are asked to *express* their knowledge on ethical decision-making and the concepts that guide them, they may lack the ability to reflexively draw the connections between these concepts. If this ethical decision-making knowledge is successfully introduced to the students, their comprehension of professional ethics within engineering may be greatly improved. Growing this relationship between common/personal ethics and professional ethics is something that engineering education curricula should be striving for when producing professional engineers that live up to the standards set by the NSPE.

### Correlation of DIT-2/EERI Scores and Concept Map Scores

To investigate the extent to which there is a correlation between conceptualizations of ethical decision-making (concept maps) and performing ethical reasoning (EERI or DIT-2), Spearman rank correlations were determined between the DIT-2 and Concept Map Scores, and between the EERI scores and the Concept map scores for each individual who had successfully completed both assessments (n = 54)—Tables 7 and 8 below. Spearman Rank Correlation assesses how well the relationship between two variables can be described using a monotonic function. Spearman correlations of + 1 or − 1 indicate perfect monotonicity (a strong correlation in either increasing (+ 1) or decreasing (− 1) monotonic trend) and a value near zero indicates no correlation (i.e., the two variables are orthogonal). Comparing the DIT-2 and EERI to the Concept Map scores, the total correlation scores for both traditional and holistic scoring systems indicate essentially no correlation, with no Spearman’s |ρ|≥ 0.2. This result is very interesting from the viewpoints of both educators and education assessment—namely that performance on the DIT-2 or EERI is likely not an indicator of concept map score, therefore implying that a first-year engineering student’s ability to perform ethical reasoning—on the EERI or DIT-2 test at least—is not well-informed by their abstract understanding of ethical decision-making concepts *prior to formal ethics education*.

**Table 7:** Spearman rank correlations ($\rho$) between DIT-2 scores and concept map scores

n = 54	Traditional scores					Holistic rubric scores			
DIT-2 scores	NC	NH	HH	NCL	Total	Comprehension	Correctness	Organization	Total
P	0.122	0.219	− 0.171	− 0.137	− 0.182	− 0.186	0.007	0.006	− 0.057
N2	0.139	0.175	− 0.003	− 0.008	− 0.043	− 0.124	0.031	0.098	0.008

**Table 8:** Spearman rank correlations ($\rho$) between EERI scores and concept map scores

n = 65	Traditional scores					Holistic rubric scores			
EERI scores	NC	NH	HH	NCL	Total	Comprehension	Correctness	Organization	Total
P	− 0.155	− 0.006	− 0.028	0.076	0.017	0.029	− 0.006	0.114	0.075
N2	− 0.115	0.009	− 0.055	0.074	0.030	0.043	0.015	0.116	0.097

## Conclusions

In this study, the baseline ethical reasoning capability, as gauged by the DIT-2 & EERI, was measured for a large group (N_DIT-2_ = 440, N_EERI_ = 425) of first year engineering students. In answer to RQ1: the baseline DIT-2 P/N2 scores of first-year engineering students are largely indistinguishable from the general population at this level of education. Likewise, EERI P/N2 scores, which in general are *numerically larger* by about 20 points than DIT-2 scores, are also similar to those previously determined in a small scale study on first-year engineers (where the EERI showed negligible change pre-to-post intervention (Cimino & Streiner, 2018). When asked to complete Concept Maps on ethical decision-making, a large variation was found in traditional scores (Traditional Score = 49.15 ± 39.40), with students focusing heavily on concepts known, while not recognizing the links between concepts. In holistic terms, comprehension and organization scores of the concept maps were weak (both < 2 out of 3), with average correctness being moderate (~ 2 out of 3). The low organization scores are in a large part due to the lack of crosslinks that illustrate the interrelation of concepts.

Perhaps the most surprising and interesting result of this study is in regard to the nature of the relationship between ethical reasoning and ethical decision-making concept knowledge among FY engineering students (RQ2). It has been found that FY engineering student concept maps generally have no bearing whatsoever on student DIT-2/EERI scores. The near-zero correlations of DIT-2 and EERI scores with concept map parameters suggest knowing about ethical concepts and performing ethical reasoning in ethical dilemmas may draw on different cognitive and emotional skills and information, and perhaps rely on different thought processes as well. This would not be surprising from a situated cognition framework. Since the situated view would contend that both ethical knowledge and ethical decision-making emerge within context, when the context changes from brief scenarios to the concept mapping task (without applied context), the situated thinking that emerges would be different. We would further contend that ethical decision-making in richly contextualized scenarios (such as immersive virtual reality or enacted role playing) would be equally different, but yet have greater invariance with real-world engineering decision-making than either the concept mapping task or the text-based scenarios task.

When asked to produce concept maps without a scenario context, students may not be using purely logical conceptual reasoning and may not even be aware of the reasoning they are using to construct the abstract concept maps. Instead, they may be relying on thought processes used in their personal lives (normative social and personal conceptions of ethical behavior), as well as their preconceived notions of how an engineer should perceive and act as a professional. Equally plausible is the possibility that they may not be applying reasoning at all—rather, they may be making judgements intuitively or instinctively, without recourse to reason. An additional factor that cannot be overlooked is that of response bias, i.e., they may be performing the task with the intentions of typical classroom assignments, seeking to produce the instructor-approved “correct” concept map response. A situated cognitive view would suggest that professional engineering practitioners would typically be influenced more by industry and societal norms including codes of ethics than they would be by personal ethics from outside their work context. This would be determined as much by the context of the decision as it would be by the engineer’s abstract knowledge and prior experiences.

We would suggest then that “engineering ethical reasoning” describes tasks in which (future) engineers are asked to draw from abstract concepts of ethical principles and apply them from a third-person perspective to one or more imaginary scenarios (as in the DIT-2 and EERI). Likewise “ethical decision-making knowledge” would describe what students produce on tasks like our concept mapping assignment, in a context that lacks any details of an applied engineering authentic context(s). Finally, we would describe our playful learning approaches (Streiner et al., 2021) that attempt to establish realistic contexts that situate students as engineering professionals making authentic ethical decisions, as “richly situated ethical decision-making”. We posit that such “richly situated ethical decision-making” most closely resembles real-world behavior from an ecological psychology perspective, wherein behavior emerges in context as an interaction of an intentional agent and an information-rich environment.

Based on our current work, several areas of inquiry arise for potential future study. In particular, the observed differences in concept maps and DIT-2/EERI scores from pre to post must be investigated with regard to specific ethics education interventions. Likewise, the effects of the ethics interventions on the instrument scores and concept maps could be investigated by employing qualitative methods such as discussion groups, interviews, think-alouds etc. The results of which could then inform traditional and alternative pedagogies, such as playful learning, to incorporate situated, authentic and contextually rich ethical decision-making strategies that support agency and experience for undergraduate engineering students.
